# Re-routing photosynthetic energy for continuous hydrogen production in vivo

**DOI:** 10.1186/s13068-019-1608-3

**Published:** 2019-11-11

**Authors:** Oren Ben-Zvi, Eyal Dafni, Yael Feldman, Iftach Yacoby

**Affiliations:** School of Plant Sciences and Food Security, The George S. Wise Faculty of Life Sciences, Tel Aviv University, Ramat Aviv, Tel Aviv, 69978 Israel

**Keywords:** *Chlamydomonas reinhardtii*, Hydrogen production, Hydrogenase, Superoxide dismutase, Fusion protein

## Abstract

**Background:**

Hydrogen is considered a promising energy vector that can be produced from sustainable resources such as sunlight and water. In green algae, such as *Chlamydomonas reinhardtii*, photoproduction of hydrogen is catalyzed by the enzyme [FeFe]-hydrogenase (HydA). Although highly efficient, this process is transitory and thought to serve as a release valve for excess reducing power. Up to date, prolonged production of hydrogen was achieved by the deprivation of either nutrients or light, thus, hindering the full potential of photosynthetic hydrogen production. Previously we showed that the enzyme superoxide dismutase (SOD) can enhance HydA activity in vitro, specifically when tied together to a fusion protein.

**Results:**

In this work, we explored the in vivo hydrogen production phenotype of HydA–SOD fusion. We found a sustained hydrogen production, which is dependent on linear electron flow, although other pathways feed it as well. In addition, other characteristics such as slower growth and oxygen production were also observed in Hyd–SOD-expressing algae.

**Conclusions:**

The Hyd–SOD fusion manages to outcompete the Calvin–Benson cycle, allowing sustained hydrogen production for up to 14 days in non-limiting conditions.

## Introduction

Hydrogen (H_2_) is a potential clean energy carrier that can replace fossil fuels when produced from renewable resources [1–3]. Photobiological H_2_ production, carried out by most green microalgae, is regarded as a promising clean and efficient source for this valuable commodity [4–6]. These photosynthetic organisms can dispose surplus electrons generated under specific conditions (e.g., anaerobiosis) [7] by expressing hydrogenases–metalloenzymes that can reversibly reduce protons to molecular H_2_ [8]. One such microalga is the well-studied eukaryotic microalgae *Chlamydomonas reinhardtii,* for which the H_2_ production process was thoroughly investigated [9–12]. *C. reinhardtii* expresses two [FeFe]-hydrogenase isoenzymes, HydA1 and HydA2, which are encoded in the nucleus, and subsequently transferred to the chloroplast stroma [13]. Activation of the hydrogenases requires the assembly of a di-iron subsite by a set of maturases (HydE/HydF and HydG), which are strongly induced upon anoxia [14]. HydA1/2 can serve as an electron sink for fermentation processes in dark, anoxic conditions [15, 16]. They are also thought to allow electron transport following dark anoxia, to build up a sufficient proton motive force for ATP production before electrons are re-directed towards CO_2_ fixation [17]. However, H_2_ production is a short-lived sink since molecular oxygen (O_2_) generated by water splitting will eventually inactivate HydA1/2 irreversibly [18–20]. Sulfur deprivation has been widely used to cope with the O_2_ sensitivity of HydA1/2, as it results in lowered levels of photosystem II (PSII) activity, leading to anoxia and sustained H_2_ production [21, 22]. Alternatively, recent protocols suggest applying chemical scavengers or using photosystem ratio imbalance to maintain anoxia in H_2_-producing cultures [23–25].

HydA1/2 are reduced by the mobile electron mediator, ferredoxin (Fd), which distributes electrons from the membrane complex photosystem I (PSI) [26]. Notably, H_2_ production is a minor sink for the electrons leaving PSI. The most prominent sink is CO_2_ fixation by the Calvin–Benson–Bassham (CBB) cycle, via the ferredoxin–NADPH oxidoreductase (FNR). FNR oxidizes Fd to create low-potential reductant (NADPH) that is essential for CO_2_ fixation. Since it has a vital role in carbon fixation, FNR evolved to be a major acceptor of the linear electron flow (LEF). With high abundance and stronger affinity for Fd [27, 28], FNR can easily outcompete HydA1/2. In addition, direct binding to PSI was also suggested as a mechanism for FNR superiority—by increasing the access of FNR to reduced Fd [29, 30].

Hydrogenase’s high sensitivity to O_2_ has been considered the most challenging limitation on algal H_2_ production [31]. However, in recent years several studies highlighted the role of the CBB cycle in inhibiting H_2_ production even before this inactivation takes place. Milrad et al. observed that under non-limiting conditions (TAP media), C. *reinhardtii* light-driven H_2_ production decays within the first 2 min following dark anoxia despite having an active pool of HydA1/2. Hence, H_2_ production is inhibited by competition with CBB cycle prior to O_2_ inactivation [32].

Recent alternative production methods, aiming to bypass the electron loss for CBB cycle, further support this observation. Nagy et al. used media lacking CO_2_ to deprive CBB cycle rendering it inactive [24]. Kosurov et al. employed a light scheme composed of fluctuations between dark and light, thus preventing the proper initiation of CBB cycle [33]. In both methods, sustained H_2_ production was observed for several days, whereas O_2_ was consumed by either chemical absorbent or respiration. A different approach to bypass the electron loss is to employ synthetic biology. Instead of directly inhibiting CBB cycle, HydA can be modified to improve its competitive ability. This could allow HydA to outcompete CBB cycle and dominate the electron pool without the need for special treatment. In this regard, we previously showed that a fusion of Fd with HydA shifts electron flow favorably towards H_2_ production rather than the FNR pathway [29]. Based on this approach, we developed a fusion of HydA to Fe-SOD (Hyd–SOD). SODs are a family of metalloenzymes which functions in the elimination of oxygen radicals in all cellular compartments [34]. *C. reinhardtii* thylakoid stroma contains the Fe-SOD type [35] and, therefore, this form was chosen as a fusion partner. HydA high susceptibility to O_2_ led us to initially hypothesize that SOD might protect HydA under aerobic stress. However, in vitro experiment conducted with purified proteins showed an unexpected enhancement of HydA activity by SOD, both in the presence and absence of oxygen. Furthermore, Hyd–SOD fusion showed remarkable capabilities in reconstituted photosynthetic experiments [36]. Although the exact mechanism was unknown, we believed the phenomenon was intriguing enough for further study. Thus, in this study we analyzed the Hyd–SOD fusion protein in vivo, focusing on its effect on C. *reinhardtii* H_2_ production under non-limiting conditions.

## Methods

### Transformation, screening and immunoblot analysis

*Chlamydomonas reinhardtii* codon-optimized sequence (GeneArt), coding for the fusion protein Hyd–SOD, was inserted into pChlamy_1 expression vector (GeneArt). The fusion sequence was cloned under the Hsp70A-RbcS2 promoter with an Fd transit peptide sequence for chloroplast delivery [37]. The plasmid was transformed by electroporation to the nucleus of *HydA1–HydA2* double mutant [38] (DM) according to GeneArt Chlamydomonas Engineering Kit protocol (Life Technologies). For positive clone screening, algae were overlaid with engineered H_2_-sensing *R. capsulatus* and left overnight [39]. The plates were then scanned using the Fuji FLA-5100 fluorescence imager. A 473-nm laser was used for excitation, whereas 510- and 665-nm filters were used for quantifying GFP luminescence and chlorophyll density, respectively. Genomic DNA isolation was preformed using E.Z.N.A.^®^ SP Plant DNA Kit (Omega Bio-tek). Immunoblot analysis was used for protein expression verification according to Eilenberg et al. [37]. Whole cell protein extraction was preformed according to Rühle et al. [40].

### Cell cultivation

Inoculums were cultivated in 50 mL TAP (Tris/acetate/phosphate) medium [41] kept in Erlenmeyer flasks capped with a silicone sponge. Cells were grown under constant irradiation of 100 µmol photons m^−2^ s^−1^ at 24.5 °C with stirring until they reached early log phase (2–5 μg[Chl] mL^−1^). Chlorophyll (Chl) was extracted and measured according to Jeffrey and Humphrey [42]. The cells were then centrifuged at 3300*g* for 2 min and resuspended in a fresh medium.

### Methyl viologen activity measurements

2 mL cell suspension (50 µg[Chl] mL^−1^) was flushed with argon gas (Ar) and incubated for 2 h in the dark while shaking (80 RPM). Glucose oxidase (40 units mL^−1^), catalase (40 units mL^−1^), and glucose (20 mM) were added for complete anoxia. Chemical reaction buffer [43] 3× stock (for final concentration of 100 mM Tris–HCl, pH 7.2, 1 M NaCl, 10 mM methyl viologen, 20 mM sodium dithionite, and 0.2% [V/V] Triton X-100) was prepared in an anaerobic glove box (H_2_/N_2_). The buffer was sealed in a vial and flushed with Ar for 10 min to remove residual H_2_ traces. For HydA activity measurements, 1 mL reaction buffer was added to the cell suspension. The vials were then incubated at 50 °C in a water bath, while 500 µL of headspace gas was drawn in 6-min intervals. The concentration of H_2_ in the vial’s headspace was measured by a Hewlett-Packard 5890 Series II gas chromatograph. For each sample, the chemical activity rate of the active HydA was determined in µmol H_2_ mg[Chl]^−1^ h^−1^.

### Oxygen measurements and electron transfer rate (ETR)

Cells were resuspended to 15 µg[Chl] mL^−1^ in fresh TAP. For O_2_ consumption, samples were directly transferred to FireStingO_2_ OXVIAL4 respiration vial (Pyro science) and kept in the dark. For net O_2_ evolution, samples were flushed with N_2_ for 5 min to remove oxygen, and then transferred to 5 mL vial fitted with FireStingO2 OXROB3 probe (Pyro science). Following a 10-min dark period, light was switched on and the irradiance was increased every 5 min. Red actinic light was supplied by a Dual-Pulse Amplitude Modulated Fluorometer (DUAL-PAM-100, Walz). The module used was DUAL-DB. ETR measurements utilized the same setup, except for using an open cuvette to maintain aerobiosis. A saturating pulse (3000 µmol photons m^−2^ s^−1^) was used at each irradiance level for quantum yield determination. The ETR was calculated according to

1 $${\text{ETR}}\, = \,Y\left( {\text{II}} \right)*{\text{ PAR}}*0.5*{\text{qA,}}$$

where *Y*(II) is the effective quantum yield of PSII during a light period (*F*/Fm′), PAR is the photosynthetically active radiation (µmol photons m^−2^ s^−1^ in the 400–700 nm range), qA is the absorbed light coefficient and 0.5 is a constant corresponding to a 1:1 ratio between PSII and PSI [44–46].

### Short-term H_2_ production

QMS 200 M1 (Pfeiffer Vacuum) membrane inlet mass spectrometer (MIMS) was used to record gas exchange in vivo. 5 mL of cells was resuspended to 15 µg[Chl] mL^−1^ in a medium containing TAP and 50 mM HEPES at pH 7.8. The culture was then incubated for 2 h in the dark in a sealed 5-mL quartz cuvette. Glucose oxidase (40 units mL^−1^), catalase (40 units mL^−1^), and glucose (20 mM) were added for complete anoxia. For each experiment, the cuvette was fitted into a metabolic chamber (Optical unit ED-101US/MD, Walz) which kept the sample at 27 °C during the experiments. The cuvette was exposed to 180 µmol photons m^−2^ s^−1^ red actinic light supplied by a Dual-Pulse Amplitude Modulated Fluorometer (DUAL-PAM-100, Walz). The modules used were DUAL-DR and NIR. Light intensity was determined by a Walz light detector (model US-SQS/L) attached to a Li-250A light meter (LI-COR Biosciences). If stated, DCMU (200 µM), DBMIB (20 µM) or GA (10 mM) was added 10 min prior to light exposure. The masses of H_2_, N, O_2_ and Ar were repeatedly measured with 3-s interval per mass. O_2_ trace was normalized to the Ar trace to compensate for the continuous removal of the measured gas by the vacuum line [47]; H_2_ signal was normalized and measured as described [48].

### Long-term H_2_ production assay

1 L of algal culture was grown in normal conditions (see “Cell cultivation”) to high density. The cells were harvested (4000×*g*, 5 min), resuspended to 10 µg[Chl] mL^−1^ in fresh TAP with 5 mM Na_2_SO_3_ [23] and transferred to 1-L BlueSens bioreactors. The bioreactors were kept at 27 °C with constant stirring. Following the transfer to the BlueSens system, the cultures were flushed with N_2_ for 2 min and subsequently kept in the dark for 2 h to achieve anaerobic conditions. At this point, light (LED, 180 µmol photons m^−2^ s^−1^) was turned on and both O_2_ and H_2_ were measured using thermal conductivity (TCD) sensors. Gas output was monitored using milligascounter mgc-1 pmma (Ritter) attached to the bioreactor headspace. If stated, 200 µM DCMU was added through a septum sealed sampling valve, or 2 mL cell suspension was drawn for starch measurements. Starch content was determined using Megazyme’s Total Starch Assay Kit (AA/AMG).

### Growth curve measurements

Aerobic growth curves were obtained using Multi-Cultivator MC 1000-OD (Photon Systems Instruments). Cells were inoculated to 0.1 *A*_680nm_ and cultivated under 27 °C, 180 µmol photons m^−2^ s^−1^ light with constant air bubbling. Cell density was measured as *A*_680nm_ in 10-min intervals. Anoxic growth was measured in 1-min intervals using Hamilton Dencytee sensor (880 nm) attached to the BlueSens bioreactor.

## Results

### Engineering of Hyd–SOD-expressing clones

**Fig. 1:**
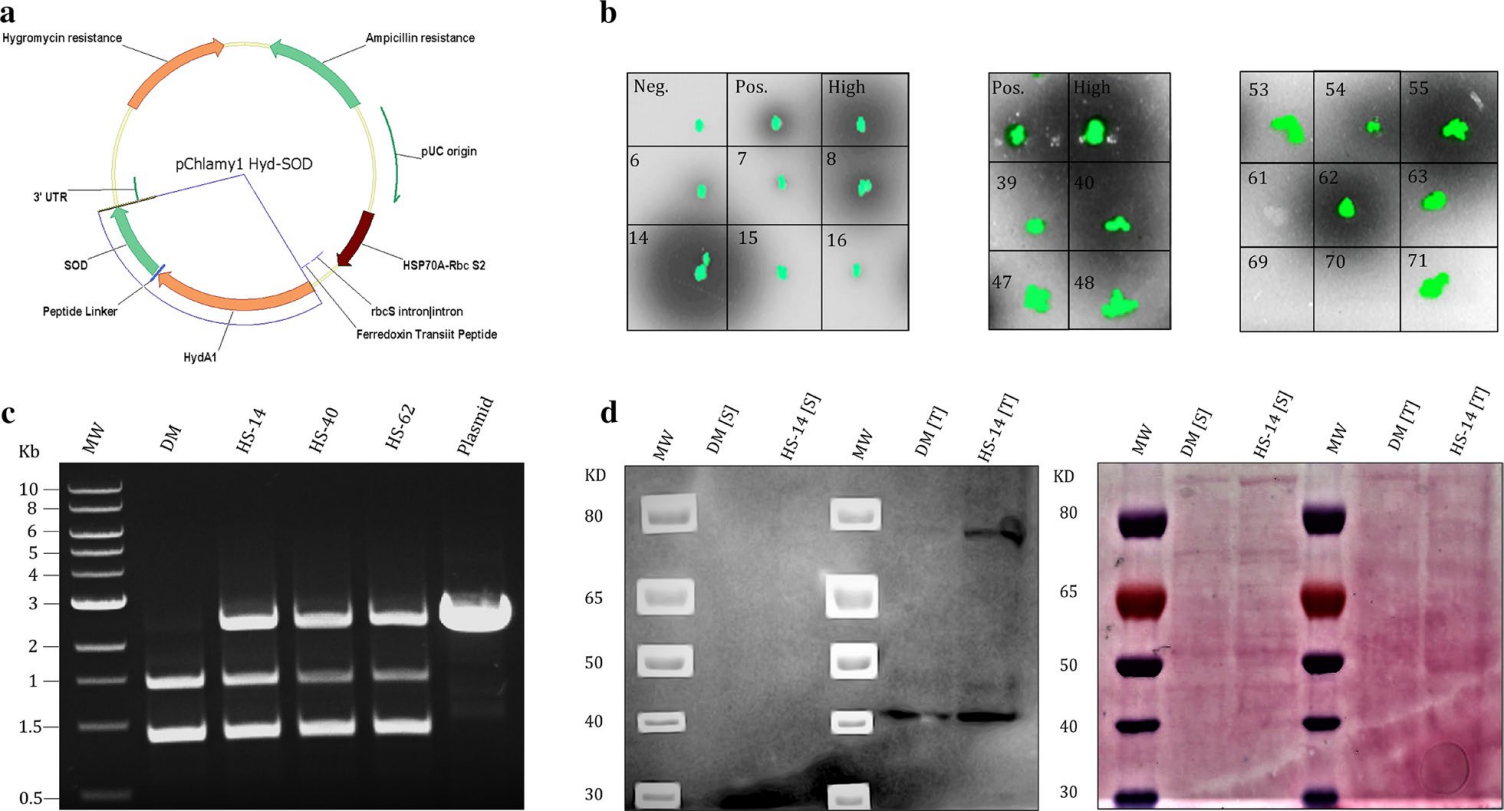
Construction and screening of HS mutants. **a** Plasmid vector map of pchlamy1 Hyd–SOD. **b** *R. capsulatus* screen (Rhodo assay) for HydA–SOD-expressing clones. Algae chlorophyll fluorescence is green whereas dark halation represents GFP produced by *R. capsulatus* due to H_2_ presence. The WT strain CC-124 (High), Fd–HydA-expressing clones (Pos.) and DM (Neg.) clones were used as positive, transformant reference and negative controls, respectively. Numbers represent HS clones. **c** PCR analysis of genomic DNA from DM, HS-14, HS-40 and HS-62 cells. Primers for pChlmy1 rbcS promotor (forward) and the 3′UTR (reverse) were used and the expected amplified chain length is 2400 bp. **d** Immunoblot analysis for the detection of Hyd–SOD expression. Soluble fraction [S] and whole cell protein extraction [T] from DM and HS-14 were loaded on 4–12% Bis–Tris PAGE (Life Technologies) and probed with rabbit polyclonal HydA1/2 antibodies and ponceau red

The Hyd–SOD gene was codon optimized, cloned into GeneArt pchlamy1 vector (Fig. 1a) and transformed by electroporation into the nucleus of DM [38]. This procedure integrates the DNA strand into the genome by non-homologous end joining, resulting in unpredicted locations of the new insert in the genome. This causes a ‘position effect’, in which expression levels vary between different clones transformed with the same DNA fragment [49]. Therefore, colonies were initially screened for H_2_ production using the *Rhodobacter encapsulates* (Rhodo) assay (Fig. 1b) [39, 50]. Since the Rhodo assay only provides a rough estimation for the expression level of the enzyme, selected clones were further analyzed by the methyl viologen (MV) assay. The MV assay measures the kinetics of H_2_ production and, therefore, can accurately quantity the active pool of the enzyme, (i.e., the mature enzyme pool) [37, 43]. To induce maturation, cultures were incubated in the dark under mild room temperature conditions. Following dark induction, the activity of the fusion was observed to be stable for several days (Additional file 1: Figure S1). Surprisingly, despite having significantly large halos in the Rhodo assay, most of the Hyd–SOD-expressing clones (HS clones) had low abundance of active protein (Fig. 1b, Table 1). Notably, even the highest expressing clone, HS-14, showed an order of magnitude less active protein than a complement strain expressing native HydA1 gene (HydA1+), in the parental DM. The low protein abundance in HS clones also made it difficult to detect the fusion protein using western blot. Even though the complete gene was successfully inserted into the genome (Fig. 1c), clear visible protein band in the correct size (~ 80 KDa) appeared only in the highest expressing HS-14 clone (Fig. 1d). Interestingly, this band could not be detected in the soluble fraction, despite the soluble nature of the fusion protein [36]. For further analysis, we selected three clones expressing the Hyd–SOD fusion protein at several levels: HS-14 (high), HS-40 (medium) and HS-62 (low) (Table 1). Thus, by exploiting the position effect, we were able to measure the correlation between fusion abundance, photosynthetic parameters and H_2_ production.

**Table 1:** Estimation of Hyd–SOD abundance by the analysis of active HydA

Strain	Active enzyme mean ± SE, µmole[H_2_] (mg[Chl] × h)^−1^
W.T [cc124]	104.6 ± 14.4
W.T [D66]	37.68 ± 8
HydA1+	34.57 ± 8.93
HS-1	0.02 ± 0.01
HS-14^a^	2.42 ± 0.54
HS-18	0.03 ± 0.02
HS-24	0.1 ± 0.02
HS-40^a^	0.62 ± 0.05
HS-52	0.12 ± 0.02
HS-55	0.06 ± 0.02
HS-62^a^	0.17 ± 0.03
HS-73	0.19 ± 0.03
HS-100	0.08 ± 0.03

^a^Selected clones

### Photosynthetic parameters

**Fig. 2:**
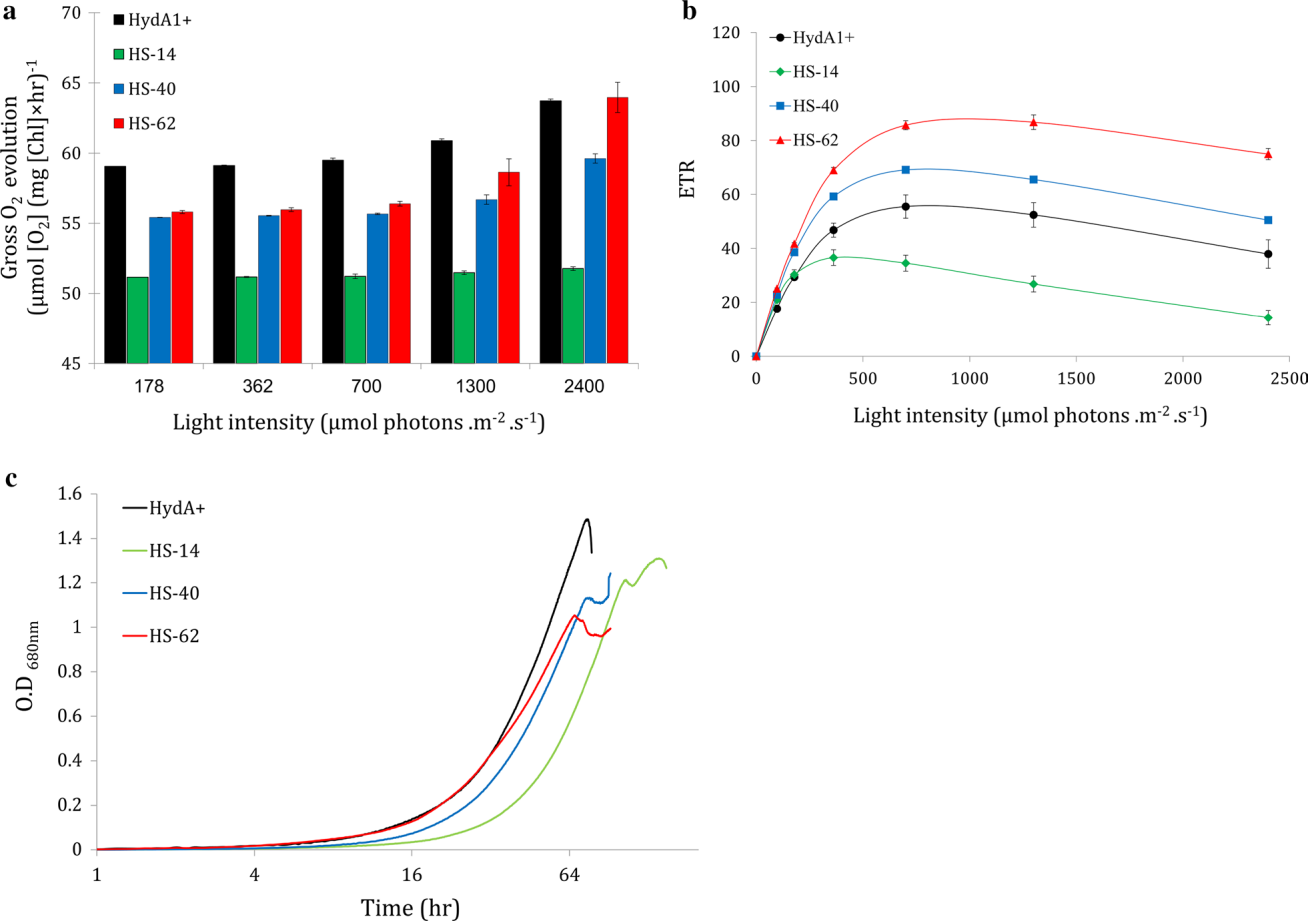
Photosynthetic parameters of HS mutants. **a** Gross O_2_ evolution in response to increased irradiance of HydA1+, HS-14, HS-40 and HS-62 (black, green, blue and red, respectively). Dissolved O_2_ evolution was measured in a 5-mL vial fitted with Pyroscience FireSting O_2_ OXROB3 probe, and OXVIAL4 respiration vial. Error bars are in SE (n = 3). **b** Chlorophyll fluorescence measurement. ETR in response to increased irradiance of HydA1+, HS-14, HS-40 and HS-62 (black, green, blue and red, respectively) was measured using Walz Dual-PAM-100. Error bars are in SE (n = 3). **c** Autotrophic growth of HydA1+ and 3 HS clones: HS-14, HS-40 and HS-62 (black, green, blue and red, respectively) under aerobic conditions, measured in *A*_680nm_

In oxygenic photosynthesis, evolution of molecular oxygen is directly linked to the activity of the photosynthetic apparatus. Therefore, photosynthesis–irradiance curves of both chlorophyll fluorescence and O_2_ kinetics were conducted to better understand the physiology of the different clones. Dissolved O_2_ consumption was measured in the dark, while net O_2_ evolution was measured under increasing irradiance. Calculation of O_2_ production rates after the deduction of mitochondrial respiration show that all of the HS clones have lower O_2_ production rates compared to HydA1+ (Fig. 2a). Interestingly, O_2_ evolution followed the opposite distribution of Hyd–SOD expression level, with HS-14 having an exceptionally low O_2_ production capability, followed by HS-40 and HS-62 with the highest oxygen production rate among the HS clones. These results suggest an impaired electron transport in HS clones. Chlorophyll fluorescence and autotrophic growth in minimal media (TP) further support these observations whereas, in accordance with O_2_ measurements, HS-14 shows the lowest ETR (Fig. 2b) and growth rate (Fig. 2c). Interestingly, HS-14 clone features photoinhibition at the relatively low light intensity of 180 µmol photons m^−2^ s^−1^. Therefore, this irradiance was chosen for further analysis.

### Photosynthetic H_2_ production

**Fig. 3:**
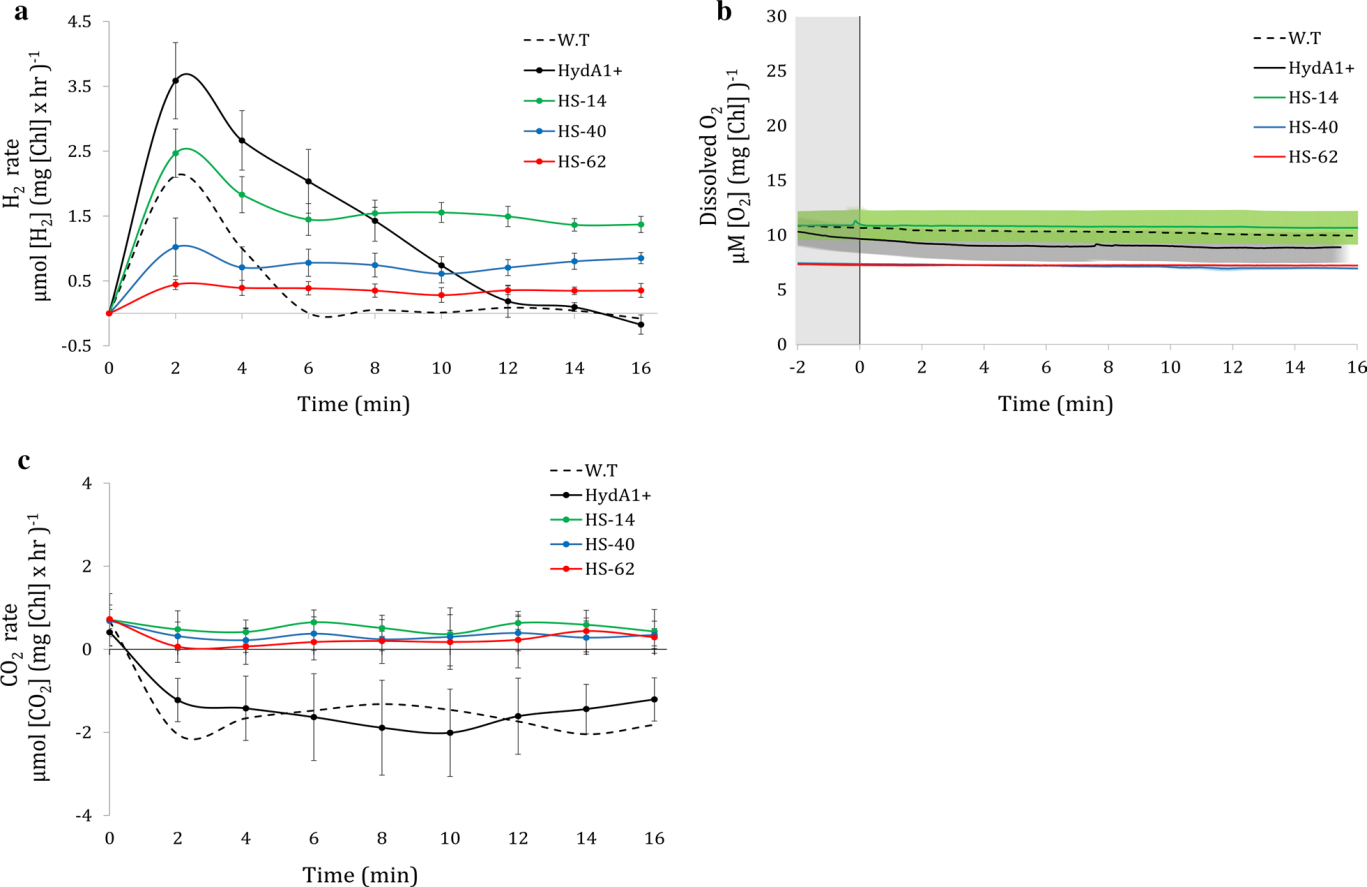
Short-term H_2_ photoproduction under anaerobiosis. H_2_, O_2_ and CO_2_ exchange was measured using a membrane inlet mass spectrometer (MIMS) in D66 wild-type (W.T, n = 3) HydA1+ (n = 7) and 3 HS clones: HS-14 (n = 7), HS-40 (n = 7) and HS-62 (n = 3) (dash, black, green, blue and red, respectively). 180 µmol photons m^−2^ s^−1^ light was switched on at t = 0. SE is represented by error bars. **a** H_2_ production rate. **b** Dissolved O_2_ concentration. SE is represented by painted area in the corresponding sample color. **c** CO_2_ exchange rate

**Fig. 4:**
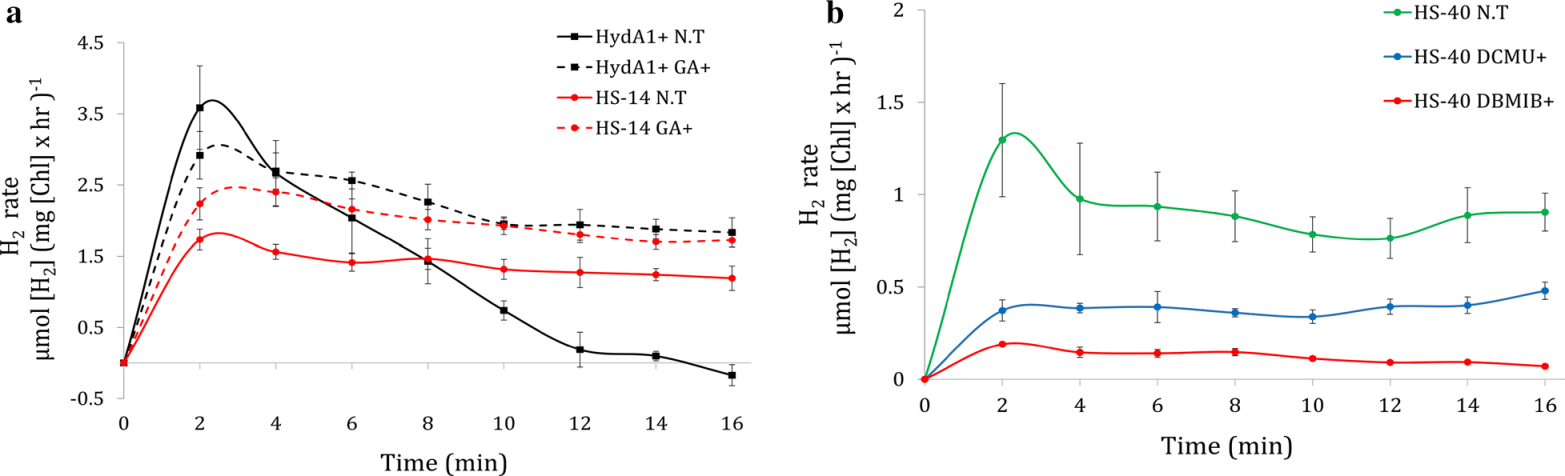
Inhibitors’ effect on H_2_ production. H_2_ production was measured using a membrane inlet mass spectrometer (MIMS). 180 µmol photons m^−2^ s^−1^ light was switched on at t = 0, whereas inhibitors were added 10 min prior. SE is represented by error bars. **a** CBB cycle inhibitor effect on H_2_ photoproduction rate. H_2_ evolution was measured in non-treated cells (NT, solid line) and GA-treated cells (GA+, dash line). HydA1+ (black, n = 4) and HS-14 (red, n = 4) were tested. **b** The effect of photosynthetic apparatus inhibitors on HS clone’s H_2_ photoproduction rate. H_2_ evolution was measured in non-treated cells (NT, n = 5), DCMU-treated cells (DCMU+, n = 4) and DBMIB-treated cells (DBMIB+, n = 3) (green, blue and red, respectively)

The photosynthetic activity of the engineered clones was analyzed using a membrane inlet mass spectrometer (MIMS, Fig. 3). Each clone was subjected to 2 h of dark incubation, during which the Hyd–SOD enzyme accumulated and matured in the presence of an active oxygen removal system (see “Methods”). Following this, the cells were exposed to an irradiance of 180 µmol photons m^−2^ s^−1^ and the dissolved H_2_, O_2_ and CO_2_ were measured for 15 min. Recently, we showed [32] that at light onset following dark anoxia, H_2_ production features a short burst of production, lasting ~ 2 min, which ceases due to competition with the CBB cycle. W.T (D66) and HydA1+ strains behaved as expected, showing a strong production rate at light onset which gradually decayed until complete cessation. HS clones, however, exhibit prolong steady linear production of H_2_, following the initial burst (Fig. 3a). Notably, O_2_ had no role in this observation, as anoxia was kept throughout the measurement by actively removing any O_2_ generated by photosynthesis (Fig. 3b), making any possible O_2_ effect on HydA activity negligible. Therefore, H_2_ production rates of the different clones are directly linked to the pool of active Hyd–SOD fusion protein within each of those clones. Indeed, H_2_ production rates follow the same pattern as the clone’s expression level. The continuous H_2_ production hints at an improved ability to compete with the CBB cycle. In addition, CO_2_ exchange at light onset shows a profound effect, in which all HS clones lack uptake of this crucial CBB cycle substrate during the measurement (Fig. 3c). To further investigate this, we added a CBB cycle inhibitor glycol aldehyde (GA). When added to HydA1+, GA altered the H_2_ production phenotype to a linear production, similar to that of the HS clones (Fig. 4a). Interestingly, while GA did not alter the H_2_ production phenotype in HS-14 clone, it had a minor effect on the H_2_ production rates. The increased rate in the presence of GA suggests that the CBB cycle is still active in the non-treated cells. Following this observation, we were interested in elucidating the electron source of the steady H_2_ production observed. We used several inhibitors to tackle this question: (i) (3-(3,4-dichlorophenyl)-1,1-dimethylurea) (DCMU), which blocks electron transfer from PSII and, thus effectively eliminates LEF. (ii) 2,5-Dibromo-6-isopropyl-3-methyl-1,4-benzoquinone (DBMIB), which inhibits electron transfer from cytochrome b_6_f and, therefore, completely deprives PSI of any electrons. When DCMU was added to HS clones, H_2_ production rate was decreased by almost 50%. The addition of DBMIB seems to have resulted in near-complete cessation of photosynthetic H_2_ production, as the observed residual production is likely due to fermentation [7] (Fig. 4b).

### Prolonged photosynthetic H_2_ production

**Fig. 5:**
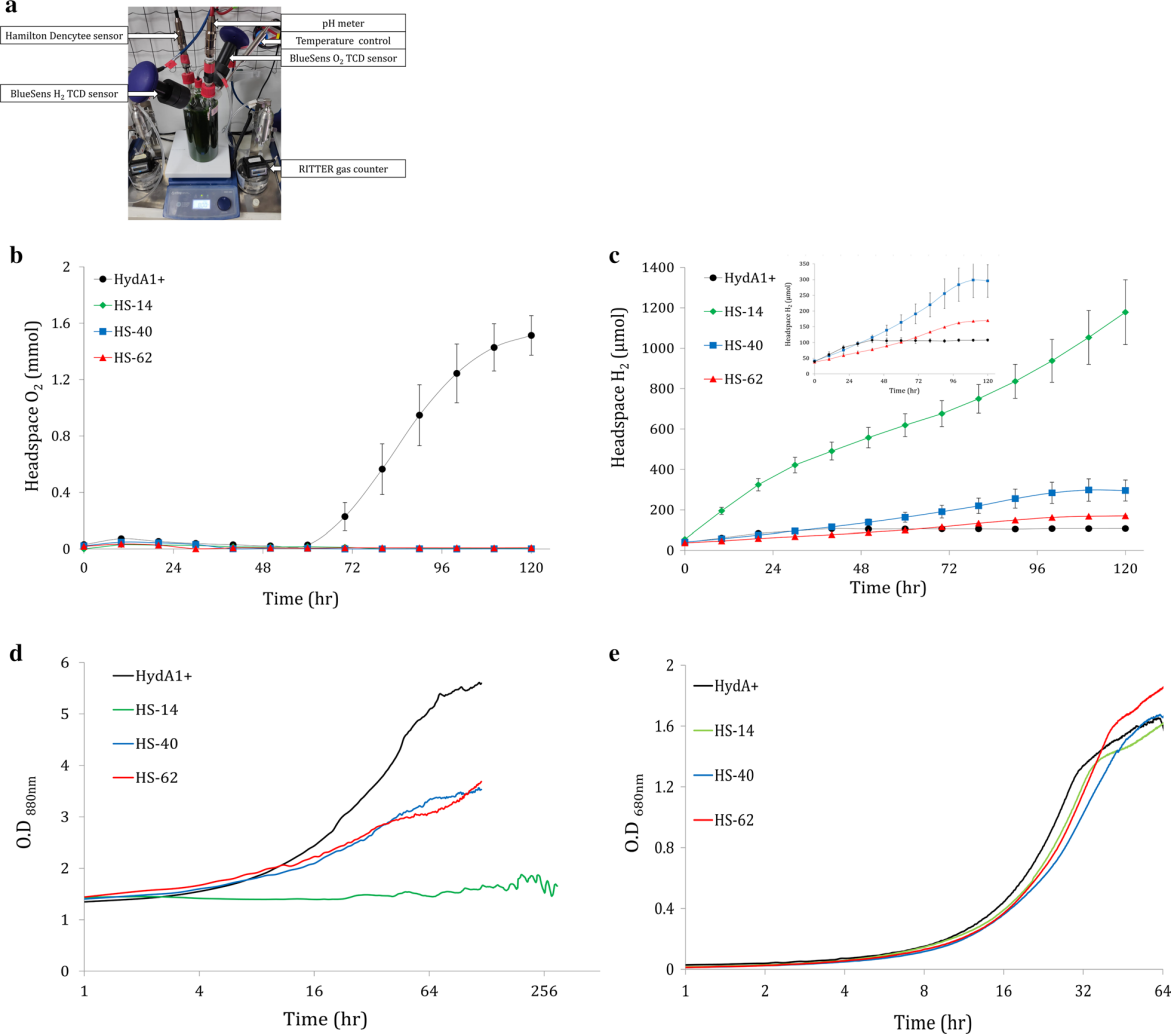
Long-term H_2_ photoproduction in 1-L BlueSens photobioreactors. **a** Picture of the BlueSens 1-L photobioreactor measuring system. The bioreactors were continuously illuminated at 180 µmol photons m^−2^ s^−1^. Cultures of HydA1+ (n = 8) and 3 HS clones: HS-14 (n = 5), HS-40 (n = 3) and HS-62 (n = 3) (black, green, blue and red, respectively) were measured. TCD sensors (BlueSens) were used to monitor. **b** O_2_ accumulation and **c** H_2_ accumulation in the bioreactor headspace. Error bars are in SE. The inset in (**c**) is a zoomed window of the blue, red and black traces. **d** Corresponding cell density of the different clones under the bioreactors anoxic mixotrophic conditions, measured in ***A***_880nm_ by Hamilton Dencytee sensor. **e** Cell density of the different clones under aerobic mixotrophic conditions, measured in ***A***_680nm_. Cultivation was performed in Multi-Cultivator MC 1000-OD under constant illumination of 180 µmol photons m^−2^ s^−1^ (n = 4)

**Fig. 6:**
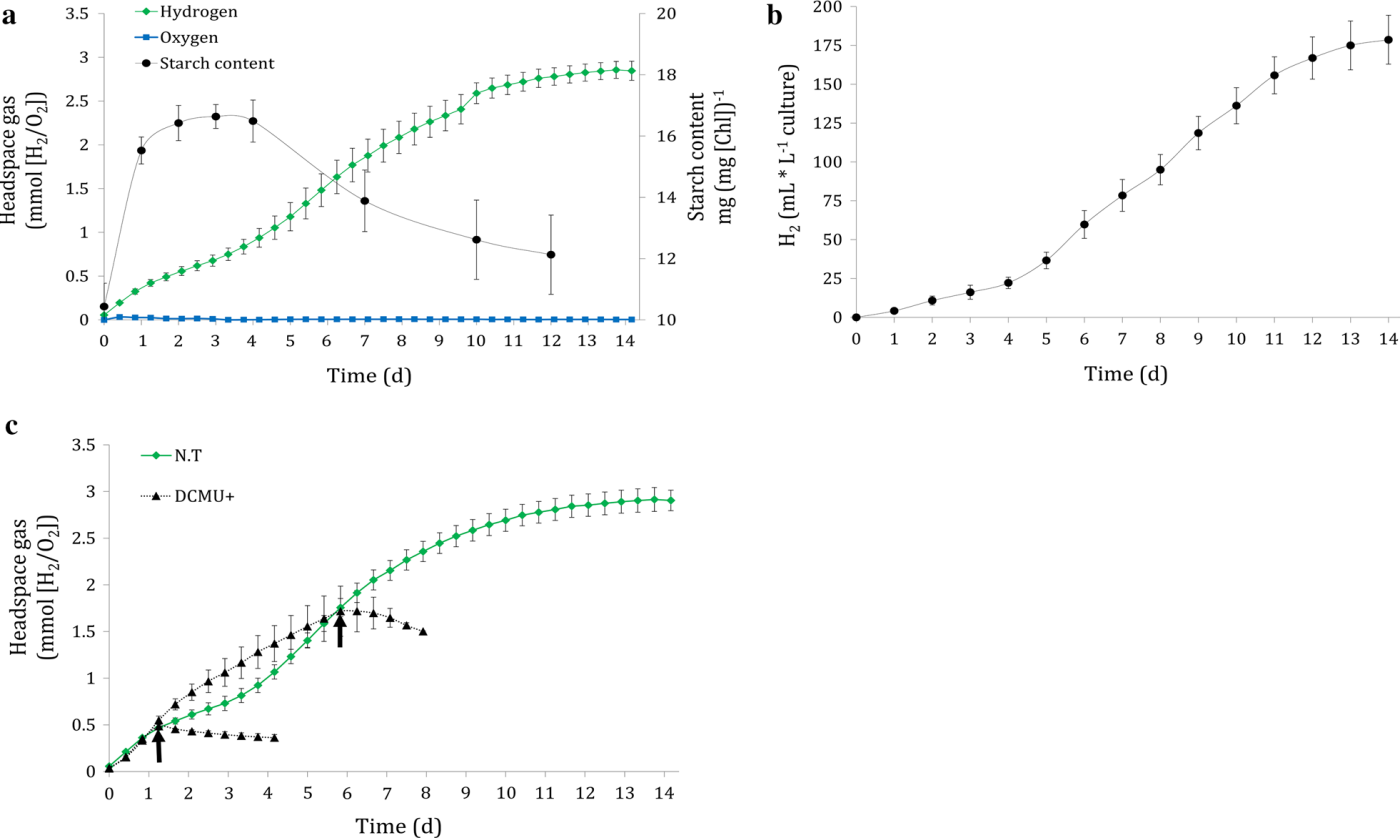
Phenotype of clone HS-14. **a** HS-14 starch accumulation during H_2_ production period. The bioreactors were continually illuminated at 180 µmol photons m^−2^ s^−1^. 2 mL samples were drawn for starch determination (black) while TCD sensors monitored H_2_ and O_2_ evolution (green and blue, respectively). **b** Gas output during starch analysis. Gas flow was measured using Ritter’s milligascounter attached to the culture headspace. Error bars are in SE (n = 4). **c** Addition of DCMU to HS-14 during H_2_ production phases. The bioreactors were continuously illuminated at 180 µmol photons m^−2^ s^−1^. Dashed black lines represent DCMU-added cultures (DCMU+), whereas the arrows indicate the DCMU addition time point. Green line represents non-treated culture (NT). Error bars are in SE (n = 4)

To estimate the longevity of H_2_ production in the HS clones, long-term experiments in TAP media were performed. We used a 1-L photobioreactor (BlueSens) designed to simultaneously monitor culture parameters such as H_2_ and O_2_ concentration, pH, OD, gas output and temperature (Fig. 5a). Importantly, under our experimental conditions (cell density of 10 µg[chl] ml^−1^ and light intensity of 180 µmol photons m^−2^ s^−1^), the oxygen uptake capacity of the engineered clones was sufficient for maintaining anaerobiosis throughout the experiment (Fig. 5b). This is extremely important since O_2_ presence is devastating for HydA activity. The exception is HydA+, which starts accumulating O_2_ after 60 h of continuous light. Notably, in HydA+, H_2_ accumulation started at time 0 and ceased after 40 h, likely due to competition with the CBB cycle, almost an entire day before oxygen started accumulating. In accordance with the MIMS results, H_2_ production rate was the highest in HS-14 followed by HS-40 and finally HS-62. All the HS clones continuously produced H_2_ for a duration of almost 5 days (Fig. 5c), apart from HS-14 which strikingly continued to produce for an additional 7 days (Fig. 6a). Interestingly, during that time, the growth of HS clones seemed to be hindered: while mixotrophic aerobically grown cultures show typical logarithmic growth phenotypes (Fig. 5e), anaerobically H_2_-producing cultures of HS-40 and HS-62 showed impaired growth for 5 days, and HS-14 growth was inhibited completely. Notably, HydA1+ still exhibits aerobic-like growth phenotype, suggesting the Hyd–SOD fusion had a role in the hindrance (Fig. 5d).

### The unique phenotype of clone HS-14

A continuous 7-day period of H_2_ production was already reported previously using various methods [51–53]. Furthermore, the recently used PSI/PSII ratio imbalance in *C. reinhardtii* C3 mutant results in a record 42 days of H_2_ production in standard TAP media (Table 2). However, the rate of H_2_ production in the C3 mutant was relatively low and not constant during these 40 days, probably due to the reduction in PSII activity [25]. Therefore, the novel capability of clone HS-14 to produce H_2_ for a period of 14 days in a high steady rate, without the need for special conditions to achieve this phenotype, is of particular interest. Analysis of starch content showed that starch was accumulated in the first 24 h and remained constant for an additional 3 days, after which a slow decrease was observed. Interestingly, starch consumption coincided with a slightly increased rate of H_2_ production (Fig. 6a). The same positive trend for H_2_ is also visible by measuring gas volume. Two phases of H_2_ production rate were observed: an initial rate of 6 mL H_2_ L^−1^ culture per day lasting for 4 days, followed by an enhanced 20 mL H_2_ L^−1^ culture per day, lasting for additional 8 days (Fig. 6b). These findings could indicate that carbohydrate could be an additional source of electron following the initial 4 days. To determine the electrons source in each phase, DCMU was added. Surprisingly, DCMU addition in both phases completely inhibited H_2_ production (Fig. 6c), suggesting that linear electron flow is a crucial source of electrons for this prolonged production.

**Table 2:** Comparison of experimental H_2_ production rates and duration by the Green Alga *Chlamydomonas reinhardtii*

Strain	Conditions\light intensity, µmol photons m^−2^ s^−1^	Max H_2_ Production rate, mL [H_2_] d^−1^ L^−1^ culture	Total duration (d)	Refs.
CC-124	Sulfur deprivation\60	11	7	Steinbeck et al. [21]
Dang 137+	Magnesium deprivation\80	10	7	Volgusheva et al. [53]
Dang 137+	Phosphorus deprivation\45	23	6	Batyrova et al. [52]
704	Low light\22	5.3	3	Jurado-Oller et al. [31]
CC-124	Light fluctuations\200	19.5	3	Kosourov et al. [33]
CC-124	CO_2_ limitation\320	50	4	Nagy et al. [24]
C3 mutant	Low light\30	3	42	Krishna et al. [25]
HS-14	Non-limiting\180	20	14	Present study

If not described by the text, H_2_ Production rate was calculated according to the publications figures. If necessary, units were converted to mL [H_2_] using PV = nRT equation

## Discussion

H_2_ production from photosynthetic organisms not only has a great potential, but also many challenges to overcome. One of the major challenges is the transitory nature of H_2_ production in green algae, which renders it unsustainable. This transitory nature was thought to stem from simultaneous O_2_ evolution and, indeed, anaerobiosis is a prerequisite for H_2_ production. Therefore, various methods were developed to eliminate the O_2_ production. Deprivation of nutrients, for example, resulting in various physiological stresses, establishes the conditions required for the induction and maintenance of long-term H_2_ production [22, 51, 52, 54]. Recently, however, Milrad et al. demonstrated that under complete anoxia, the fierce competition with the CBB cycle prevents sustained H_2_ production at light onset following dark anoxia [32]. Further evidence for this observation is provided by two new methods that support prolonged production [24, 33]. In both methods, the prolonged production is enabled by reducing the electron loss to the CBB cycle, by limiting either CO_2_ or light duration. Thus, overcoming the competition with the CBB cycle is the first hurdle in the process of engineering viable H_2_ production. In this work, we report sustained H_2_ production under non-limiting conditions via the expression of the Hyd–SOD fusion protein, demonstrating the protein’s ability to outcompete the CBB cycle.

It is well documented that steady H_2_ production can take place as long as the CBB cycle is inhibited [24, 55]. Such inhibition, if constant, is expected to manifest in a reduced growth rate due to the inhibition of carbon fixation [56]. Furthermore, a diminished CBB cycle will eventually result in overreduction of the plastoquinone pool and acidification of the lumen [57, 58]. Consequently, the overall photosynthetic activity will decelerate leading to impaired ETR and O_2_ evolution [59, 60]. Indeed, HS clones exhibit diminished rates of O_2_ evolution in accordance to the expression level of a given clone. This observation is particularly noticeable in the high expressing HS-14. Furthermore, autotrophic growth of the HS clones was also slightly impaired (Fig. 2). This is an unexpected phenotype for the fusion protein, as HydA should not remain active under aerobic conditions. Therefore, it is likely caused by the SOD moiety. Conversely, in air-grown mixotrophic cultures, the lower photosynthetic activity did not result in a slower growth rate (Fig. 5e). These findings suggest that while the inactive fusion protein might affect the CBB cycle, it is negligible under non-limiting mixotrophic conditions.

Upon HydA activation, the effect of the fusion protein becomes apparent. Normally, at light onset following dark anoxia, H_2_ production is characterized by a short burst which decays within few minutes. However, real-time MIMS measurement on HS clones showed a stable linear H_2_ production phonotype similar to the phenotype with inhibited CBB cycle in the HydA1+ complement strain (Figs. 3a, 4a). However, in contrast to HydA1+, the linear H_2_ production phenotype of HS clones is observed even when the CBB cycle is active, as was demonstrated by the addition of GA to HS-14 (Fig. 4a). Strikingly, in scaled-up 1-L photobioreactor experiments following an anaerobic incubation of 2 h, sustained H_2_ production lasted up to 14 days. The energy source for this prolonged production was mainly LEF, with some other contributions through the quinone pool, such as carbohydrate degradation, as was shown by the determination of starch content (Fig. 6a). The dominant contribution of LEF was demonstrated by the elimination of ~ 50% of H_2_ production rate following the addition of DCMU in short-term measurement (Fig. 4b) and the complete cessation of the process in the long term (Fig. 6c). This phenomenon could also explain the contradiction between the large Rhodo halos and the relatively low abundance of active protein (Fig. 1b, Table 1). Since the Rhodo assay lasts several hours, H_2_ continuously accumulated in the HS mutants, leading to larger halos in comparison to the W.T (for which high, but very short duration of H_2_ production takes place). Since the addition of an oxygen removal system effectively eliminates the effect of O_2_ on HydA activity, we hypothesize that the continuous H_2_ production of the Hyd–SOD fusion protein is due to improved ability to compete with the CBB cycle. Furthermore, the positive correlation between the expression level of the fusion protein and photosynthetic H_2_ production rate might indicate that the entire pool of active Hyd–SOD participates in the photosynthetic H_2_ production. In other words, the rate-limiting step is protein expression. Further support can be found by inspecting the anoxic growth of the HS clones during the H_2_ production phase. It seems that while the Hyd–SOD fusion protein is active under anoxia, it manages to divert electrons from the CBB cycle. This leads to a slower growth rate than the HydA1+ complement strain under anaerobiosis, especially in the high expressing HS-14 (Fig. 5d). Another intriguing feature of the HS-14 clone is its exceptionally prolonged H_2_ production, lasting for 14 days. Since DCMU addition during carbohydrate degradation phase inhibits H_2_ production (Fig. 6c), starch consumption following the initial 5 days can be interpreted as not being the source for H_2_ production, but rather as an extra energy source for vitality, allowing the cells to survive this extended anaerobic period. Indeed, the extended time in the bioreactor conditions did not result in the culture death, as inoculum taken from the bioreactor to a fresh medium was successfully regrown (data not shown).

Although much effort was invested to understand the molecular mechanism behind this phenomenon, we could not reach a detailed explanation and, therefore, the mechanism behind the continuous H_2_ production by Hyd–SOD is still largely elusive. Since immunoblot analysis showed that the protein was localized in the insoluble/membrane fraction and not in the soluble fraction as expected (Fig. 1d), we postulate that it might bound to PSI or it surrounding as suggested by Asada [61]. In addition, the impaired autotrophic growth and O_2_ evolution in all the HS clones further support possible binding. We hypothesized that binding of Hyd–SOD to PSI could interfere with FNR activity and leads for these observations. Indeed, we were able to obtain some evidence indicating a direct binding of the fusion protein to PSI; however, decisive proof could not be obtained. For example, while studying the interaction using quartz crystal microbalance and dissipation [62] did support this notion, a classical isothermal titration calorimetry [56] and pull-down assays (Additional file 1: Figure S2) could not verify these findings. In this regard, it was already suggested that PSI binding could improve H_2_ production in *C. reinhardtii* using the Fd–Hyd fusion protein [37]. However, attachment of a fusion protein to the single Fd-binding pocket [56] will likely reduce the accessibility of the binding site to free Fd; thus, the ability to reduce the HydA moiety might be affected. This possible hindrance can be overcome by fusing other partners to HydA. Indeed, Hyd–SOD clones surpass Fd–Hyd clones in both H_2_ production rate and longevity, especially considering the low active protein abundance of the Hyd–SOD clones [43].

Another possible explanation for the improved ability of the Hyd–SOD fusion protein to compete with the CBB cycle is by outcompeting soluble FNR in the stroma, for example, via improved affinity to free Fd or by stabilizing product intermediates for faster catalysis. Thus, the addition of CBB cycle inhibitor, GA, should increase the availability of reduced Fd in the stroma and result in an increased rate of H_2_ production. While such an increase was visible, it cannot be attributed to this specific hypothesis, as inhibition of the CBB cycle would result in higher H_2_ production rate anyway, either because of direct binding to PSI or because of a stroma-scavenging mechanism.

## Conclusions

Hyd–SOD fusion represents a promising pathway to overcome major challenges in H_2_ production. Using this fusion technology, we managed to engineer an algae strain that could continuously produce H_2_ for several days under non-limiting conditions. The Hyd–SOD fusion derives its electrons from water splitting. These results imply that HydA modification by fusion technology can be applied to overcome for CO_2_ fixation competition without relying on destructive ways such as nutrient deprivation.

## Supplementary information

**Additional file 1: Figure S1.** Active protein abundance during Long-term H_2_ photoproduction. Cultures of HS-14 (n = 4) were placed in BlueSens 1 L-photobioreactor measuring system. Following 2 h dark induction, the bioreactors were continuously illuminated at 180 μmol photons m^−2^ s^−1^. For hydrogenase activity measurement, 2 mL samples were drawn and a modified MV assay was used, in which the samples were directly added to the activity buffer without dark incubation (see “Method”). Error bars represent standard error. **Figure S2.** Hyd-SOD Pull down assay. Recombinant HydA and Hyd-SOD were expressed and purified as described previously (Ref). Histaged PSI was purified according to (Ref). For pull down assay, purified Histaged PSI was incubated with nickel beads, followed by 3 washing steps. Then the protein of choice was incubated with the PSI coated beads for 15 min, followed by 3 additional washing steps. The supernatant and beads containing pellet were analyze by immunoblot assay (see “Method”). PSI-represent non coated beads and PSI+ represent PSI coated beads.

## Data Availability

The datasets used and/or analyzed during the current study are available from the corresponding author on reasonable request.

## Abbreviations

HydA: algal [FeFe]-hydrogenase
SOD: algal [Fe] superoxide dismutase
Hyd–SOD: [FeFe]-hydrogenase–superoxide dismutase
H_2_: hydrogen gas
O_2_: molecular oxygen
PSII: photosystem II
Fd: ferredoxin
PSI: photosystem I
CBB: Calvin–Benson–Bassham
FNR: ferredoxin–NADP+-reductase
LEF: linear electron flow
Fd–HydA: ferredoxin–[FeFe]-hydrogenase fusion protein
PSI: photosystem-I
Hyd–SOD: [FeFe]-hydrogenase–superoxide dismutase fusion protein
DM: *HydA1–HydA2* double mutant
TAP: tris/acetate/phosphate
Chl: chlorophyll
Ar: argon gas
ETR: electron transport rate
TCD: thermal conductivity detector
MV: methyl viologen
Rhodo: *Rhodobacter encapsulates*
HS clones: Hyd–SOD-expressing clones
MIMS: membrane inlet mass spectrometer
GA: glycol aldehyde
DCMU: (3-(3,4-dichlorophenyl)-1,1-dimethylurea)
DBMIB: dibromo-6-isopropyl-3-methyl-1,4-benzoquinone
