# Supplementary material for: Re-routing photosynthetic energy for continuous hydrogen production in vivo
Source: Biotechnol Biofuels. 2019 Nov 11;12:266. doi: 10.1186/s13068-019-1608-3 (PMC6844042; doi:10.1186/s13068-019-1608-3)
Supplement: Supplementary file 1 — Additional file 1: Figure S1. Active protein abundance during Long-term H2 photoproduction. Cultures of HS-14 (n = 4) were placed in BlueSens 1 L-photobioreactor measuring system. Following 2 h dark induction, the bioreactors were continuously illuminated at 180 μmol photons m−2 s−1. For hydrogenase activity measurement, 2 mL samples were drawn and a modified MV assay was used, in which the samples were directly added to the activity buffer without dark incubation (see “Method”). Error bars represent standard error. Figure S2. Hyd-SOD Pull down assay. Recombinant HydA and Hyd-SOD were expressed and purified as described previously (Ref). Histaged PSI was purified according to (Ref). For pull down assay, purified Histaged PSI was incubated with nickel beads, followed by 3 washing steps. Then the protein of choice was incubated with the PSI coated beads for 15 min, followed by 3 additional washing steps. The supernatant and beads containing pellet were analyze by immunoblot assay (see “Method”). PSI-represent non coated beads and PSI+ represent PSI coated beads. [file 13068_2019_1608_MOESM1_ESM.pdf]

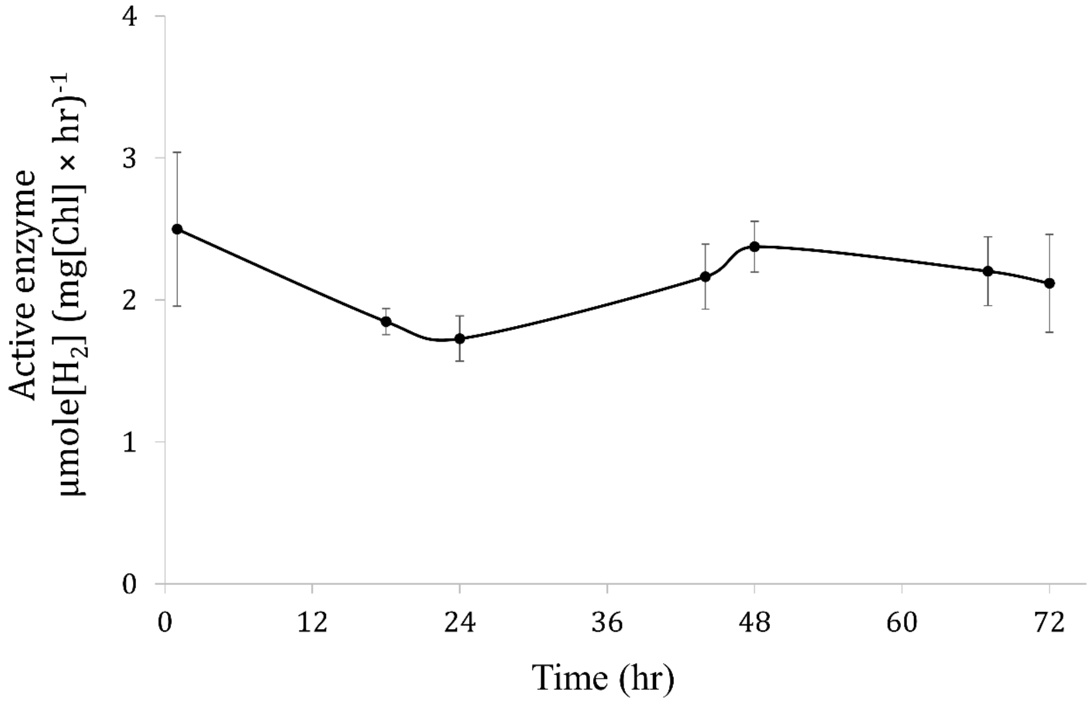

Figure S1 – Active protein abundance during Long-term H_2 photoproduction.
Cultures of HS-14 (n=4) were placed in BlueSens 1 L-photobioreactor measuring system. Following 2hr dark induction, the bioreactors were continuously illuminated at 180 µmol photons·m^{-2}·s^{-1}. For hydrogenase activity measurement, 2mL samples were drawn and a modified MV assay was used, in which the samples were directly added to the activity buffer without dark incubation (*see method*). Error bars represent standard error.

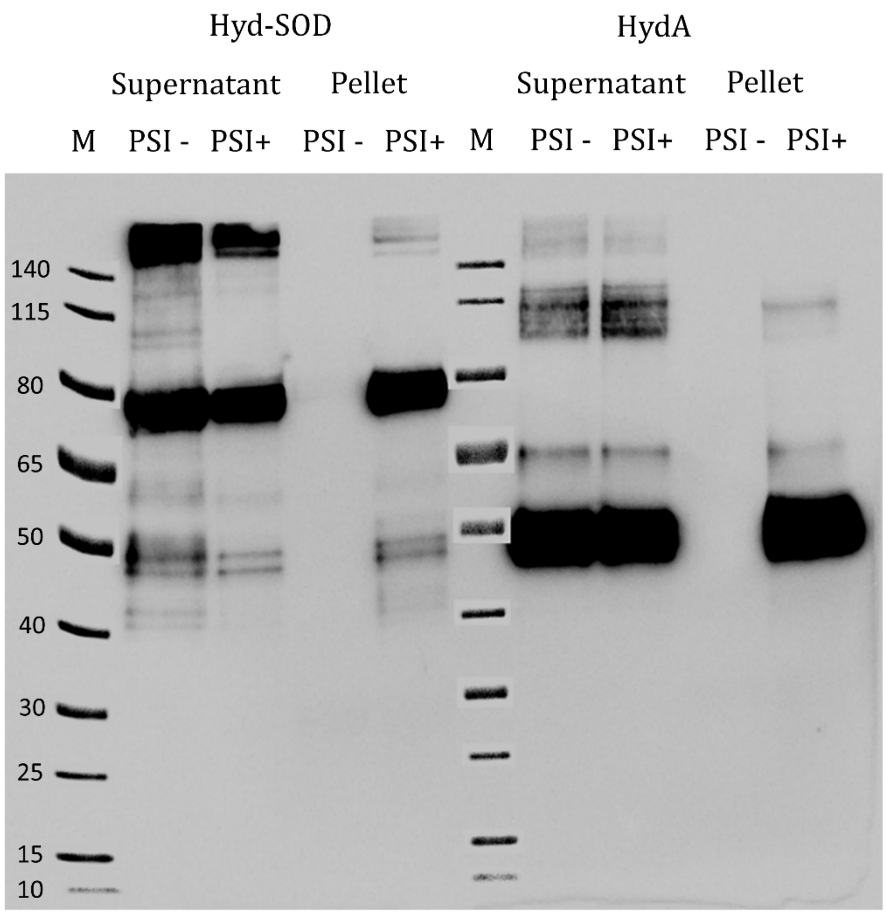

Figure S2 – Hyd-SOD Pull down assay.
Recombinant HydA and Hyd-SOD were expressed and purified as described previously (Ref). Histaged PSI was purified according to (Ref). For pull down assay, purified Histaged PSI was incubated with nickel beads, followed by 3 washing steps. Then the protein of choice was incubated with the PSI coated beads for 15 min, followed by 3 additional washing steps. The supernatant and beads containing pellet were analyze by immunoblot assay (*see method*). PSI- represent non coated beads and PSI+ represent PSI coated beads.
